# Supplementary material for: Resolving the full spectrum of human genome variation using Linked-Reads
Source: Genome Res. 2019 Apr;29(4):635–45. doi: 10.1101/gr.234443.118 (PMC6442396; doi:10.1101/gr.234443.118)
Supplement: Supplemental Material [file supp_gr.234443.118_Supplemental_longranger-2.2.zip › longranger-2.2.tar.bz2!/lr2.2-public/lib/stan/cmdstan/stan/doc/api/html/classstan_1_1mcmc_1_1fixed__param__sampler.html]

Stan: stan::mcmc::fixed\_param\_sampler Class Reference


|  |  |
| --- | --- |
|  | Stan  2.5.0  probability, sampling & optimization |


- Main Page
- Related Pages
- Namespaces
- Classes
- Files

- Class List
- Class Index
- Class Hierarchy
- Class Members

All Classes Namespaces Files Functions Variables Typedefs Enumerations Enumerator Friends Macros Pages

- stan
- mcmc
- fixed\_param\_sampler

Public Member Functions |
List of all members

stan::mcmc::fixed\_param\_sampler Class Reference

`#include <fixed_param_sampler.hpp>`

Inheritance diagram for stan::mcmc::fixed\_param\_sampler:

|  |  |
| --- | --- |
| Public Member Functions | |
|  | fixed\_param\_sampler (std::ostream \*o=&std::cout, std::ostream \*e=0) |
|  | |
| sample | transition (sample &init\_sample) |
|  | |
| Public Member Functions inherited from stan::mcmc::base\_mcmc | |
|  | base\_mcmc (std::ostream \*o, std::ostream \*e) |
|  | |
| virtual | ~base\_mcmc () |
|  | |
| std::string | name () |
|  | |
| virtual void | write\_sampler\_param\_names (std::ostream &o) |
|  | |
| virtual void | write\_sampler\_params (std::ostream &o) |
|  | |
| virtual void | get\_sampler\_param\_names (std::vector< std::string > &names) |
|  | |
| virtual void | get\_sampler\_params (std::vector< double > &values) |
|  | |
| virtual void | write\_sampler\_state (std::ostream \*o) |
|  | |
| virtual void | get\_sampler\_diagnostic\_names (std::vector< std::string > &model\_names, std::vector< std::string > &names) |
|  | |
| virtual void | get\_sampler\_diagnostics (std::vector< double > &values) |
|  | |

|  |  |
| --- | --- |
| Additional Inherited Members | |
| Protected Attributes inherited from stan::mcmc::base\_mcmc | |
| std::string | name\_ |
|  | |
| std::ostream \* | out\_stream\_ |
|  | |
| std::ostream \* | err\_stream\_ |
|  | |

## Detailed Description

Definition at line 14 of file fixed\_param\_sampler.hpp.

## Constructor & Destructor Documentation

|  |  |  |  |
| --- | --- | --- | --- |
| stan::mcmc::fixed\_param\_sampler::fixed\_param\_sampler | ( | std::ostream \* | *o* = `&std::cout`, |
|  |  | std::ostream \* | *e* = `0` |
|  | ) |  |  |

inline

Definition at line 18 of file fixed\_param\_sampler.hpp.

## Member Function Documentation

|  |  |  |  |  |  |
| --- | --- | --- | --- | --- | --- |
| sample stan::mcmc::fixed\_param\_sampler::transition | ( | sample & | *init\_sample* | ) |  |

inlinevirtual

Implements stan::mcmc::base\_mcmc.

Definition at line 21 of file fixed\_param\_sampler.hpp.

---

The documentation for this class was generated from the following file:

- src/stan/mcmc/fixed\_param\_sampler.hpp


---

[ Stan Home Page ]

*© 2011–2014,
Stan Development Team.*
